# sTNFR1 is Associated with Depressive Symptoms in Community-Dwelling Older Adults: A Logistic Regression Analysis

**DOI:** 10.1093/geroni/igaf122.1566

**Published:** 2025-12-31

**Authors:** Russell Calderon, Nada Lukkahatai, Jing Huang, Melissa Hladek, Junxin Li

**Affiliations:** Johns Hopkins School of Medicine, Baltimore, Maryland, United States; Johns Hopkins University, Baltimore, Maryland, United States; Johns Hopkins University, Baltimore, Maryland, United States; Johns Hopkins University, Baltimore, Maryland, United States; Johns Hopkins University, Baltimore, Maryland, United States

## Abstract

Inflammation has been implicated in the pathophysiology of depression, but specific inflammatory pathways may differentially contribute to depressive symptoms in older adults. This study examines the relationship between inflammatory biomarkers and depression in 91 community-dwelling older adults without dementia [Montreal Cognitive Assessment ≥17] using baseline data from a randomized clinical trial (NCT03959202). Depression was assessed using the Geriatric Depression Scale, with scores >5 indicating depression. Plasma-derived inflammatory biomarkers, including C-reactive protein (CRP), interleukin-6 (IL-6), tumor necrosis factor-alpha (TNF-α), and soluble TNF receptor-1 (sTNFR1), were Z-score normalized. A logistic regression model examined associations between biomarkers and depression, adjusting for age, sex, body mass index, and mild cognitive impairment (MCI) status. Participants were 70.2±6.2 years old, 80.2% females, 19.8% with MCI. Overall, 20.8% of participants (n = 19) were classified as having depression. In adjusted analyses, sTNFR1—a key mediator for TNF signaling—was significantly associated with depression (OR = 2.89, 95% CI [1.20, 5.60], p = 0.009), indicating that individuals with higher sTNFR1 levels had nearly three times the odds of depression. However, CRP, IL-6, and TNF-α were not significantly related to depression. These findings support sTNFR1 as a potential inflammatory biomarker linked to late-life depression, independent of demographics and cognitive status. Clinically, measuring sTNFR1 levels in routine visits may help in identifying older adults with risk for depression and informing early intervention strategies. Given its role in TNF-mediated inflammation, sTNFR1 warrants investigation as a potential target for interventions addressing late-life depression. Larger longitudinal studies should further elucidate the causal role of TNF signaling pathways in geriatric depression.

